# How to Use the Ophthalmoscope

**Published:** 1877-08

**Authors:** 


*How to use the Ophthalmoscope.* By EDGAR A. BROWN, Surgeon to the Liverpool Eye and Ear Infirmary, etc. Philadelphia: H. C. Lea. Buffalo: T. H. Butler, 800 pp. 120.

This is a brief and excellent guide for learners in ophthalmoscopy. It has numerous illustrations, and the text is very plain, and easily understood. It will have numerous purchasers.

